# Enhanced Therapeutic Efficacy of the Nanoscale Fluoropyrimidine Polymer CF10 in a Rat Colorectal Cancer Liver Metastasis Model

**DOI:** 10.3390/cancers16071360

**Published:** 2024-03-30

**Authors:** Charles Chidi Okechukwu, Xue Ma, Naresh Sah, Chinnadurai Mani, Komaraiah Palle, William H. Gmeiner

**Affiliations:** 1 Integrative Physiology and Pharmacology Graduate Program and Department of Cancer Biology, Wake Forest University School of Medicine, Winston-Salem, NC 27157, USA; cokechuk@wakehealth.edu; 2 Department of Cancer Biology, Wake Forest University School of Medicine, Winston-Salem, NC 27157, USA; 3 Department of Orthopedic Surgery, Wake Forest University School of Medicine, Winston-Salem, NC 27157, USA; xma@wakehealth.edu; 4 Department of Cell Biology and Biochemistry, Texas Tech University Health Sciences Centre, Lubbock, TX 79430, USA; naresh.sah@ttuhsc.edu (N.S.); chinnadurai.mani@ttuhsc.edu (C.M.); komaraiah.palle@ttuhsc.edu (K.P.)

**Keywords:** colorectal cancer, liver metastasis, fluoropyrimidine, replication stress

## Abstract

**Simple Summary:**

Liver metastases are a major cause of colorectal cancer mortality. To combat this, chemotherapy is widely used but generally does not result in long-term survival and causes serious side effects in many patients. We tested if a new polymeric chemotherapy, CF10, was more effective than the current drug, 5-FU, in a rat model of liver metastatic disease. Our studies proved that CF10 was better tolerated in rats than 5-FU, was more potent at decreasing the viability of rat colon cancer cells, and was significantly more effective than 5-FU at inhibiting cancer progression in a rat model of liver metastasis when administered at an equivalent dose. Based on these studies, we propose advancing CF10 into clinical trials and testing it for improved activity in human patients with colorectal liver metastases.

**Abstract:**

Combination chemotherapy regimens that include fluoropyrimidine (FP) drugs, e.g., 5-fluorouracil (5-FU), are central to the treatment of colorectal cancer liver metastases (CRLMs), a major cause of cancer mortality. We tested a second-generation FP polymer, CF10, in a CC531/WAGRij syngeneic orthotopic rat model of liver metastasis to determine if CF10 improved response relative to 5-FU. CF10 displayed increased potency relative to 5-FU in CC531 rat colorectal cancer cells based on clonogenic assay results and caused increased apoptosis, as shown using a live/dead assay. The increased potency of CF10 to CC531 cells was associated with increased replication stress, as assessed by Western blot for biomarkers of ATR/Chk1 and ATM/Chk2 pathway activation. CF10 dosed to deliver equivalent FP content as an established dose of 5-FU in rats (50 mg/kg) did not cause weight loss in WAGRij rats even when combined with ethynyl uracil (EU), an inhibitor of dihydropyrimidine dehydrogenase, the enzyme primarily responsible for 5-FU degradation in the liver. In contrast, 5-FU caused significant weight loss that was exacerbated in combination with EU. Importantly, CF10 was significantly more effective than 5-FU at inhibiting tumor progression (~90% reduction) in the CC531/WAG/Rij CRLM model. Our results reveal strong potential for CF10 to be used for CRLM treatment.

## 1. Introduction

Colorectal cancer (CRC) is the second leading cause of cancer-related mortality in men and women combined [1] (>940,000 deaths worldwide in 2020) and metastatic progression is almost exclusively responsible for all CRC mortality. Primary CRC most frequently metastasizes to the liver (colorectal liver metastasis (CRLM)) [2], with synchronous metastases in the liver occurring in 13.8–17.1% of cases and metachronous liver metastases in 7.6–15.1% of CRC [3]. Liver resection is a treatment that offers the best possibility for long-term survival in CRC patients with liver metastatic disease (40% 5-year survival) [4], but it is not achievable in >70% of cases [5]. Chemotherapy is crucial for disease management in most instances. A significant survival advantage for CRLM treatment was realized with the introduction of 5-FU chemotherapy and its modulation with leucovorin (LV), which resulted in survival benefits lasting 3–6 months [6]. The introduction of FP-based doublet chemotherapy regimens, in which 5-FU/LV is combined with oxaliplatin (FOLFOX) or irinotecan (FOLFIRI), further improved objective response rates and overall survival rates, which represent the current standard of care to improve survival [7]. While triplet therapy with FOLFOXFIRI is associated with further increased conversion to resectable disease and increased overall survival rates [8,9], this is achieved in only a limited percentage of CRLM patients because serious toxicities limit the use of this regimen to patients with a high-performance status. Thus, the development of new chemotherapies is important to further improve survival rates for CRLM patients. 

**Figure 1:**
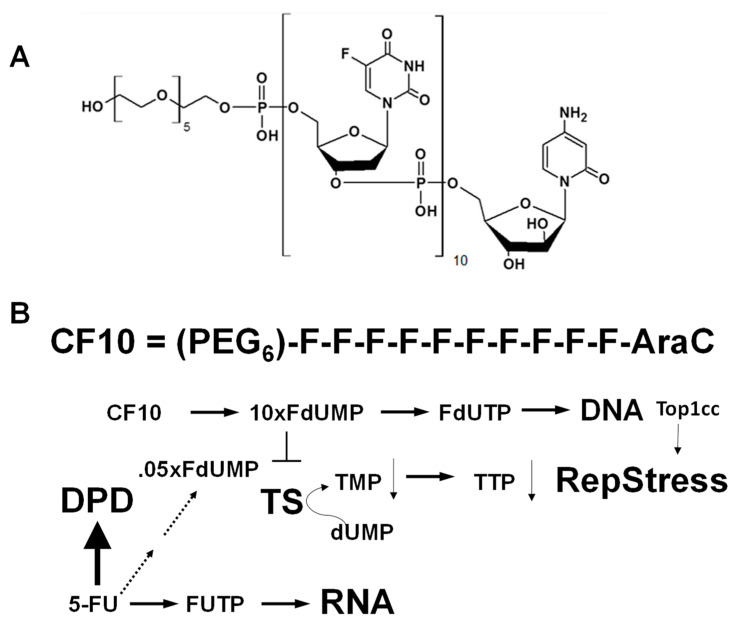
(**A**) Chemical structure of CF10; (**B**) scheme depicting the conversion of CF10 to 10× equivalents of fluorodeoxyuridylate, while only ~5% (0.05×) of 5-FU is converted to FdUMP. TS inhibition results in the depletion of TTP that causes replication fork stalling and collapse. The misincorporation of FdUTP into DNA causes the formation of DNA topoisomerase 1 cleavage complexes (Top1ccs) that stall replication forks.

To overcome the limitations of 5-FU due to inefficient TS inhibition, we developed polymeric fluoropyrimidines (FPs) that are more directly converted to fluorodeoxyuridylate (FdUMP), the TS-inhibitory metabolite of FPs. Thymidylate synthase (TS) is considered the primary molecular target for FP drugs because the more rapid proliferation of tumor cells increases cell reliance upon the de novo thymidylate biosynthesis pathway [10]. The importance of TS expression for developing resistance to 5-FU-based therapies [11] was shown in multiple studies, including a meta-analysis of 13 studies that demonstrated that elevated TS was associated with poor outcomes [12]. *TYMS* gene amplification in CRLMs was associated with shorter median survival time for patients treated with 5-FU-based chemotherapy [13]. Pre-clinical studies showed that the prototype FP polymer F10 improved antitumor activity relative to 5-FU in multiple tumor models [14,15] through a mechanism involving highly potent TS inhibition and the formation of DNA–protein crosslinks with DNA topoisomerase 1 (Top1), referred to as Top1 cleavage complexes (Top1ccs) [16,17]. We developed a second-generation FP polymer, CF10 (Figure 1), which was chemically modified to reduce exonucleolytic degradation and demonstrated that CF10 was highly effective in a mouse orthotopic model of primary colon cancer [18].

In this study, our primary objective was to test if CF10 displayed improved efficacy in a rat model of CRC liver metastatic disease. We used a modified clonogenic assay to demonstrate whether CF10 displayed improved potency relative to 5-FU in a rat colon carcinoma cell line, CC531, and we showed whether it was a potent inducer of apoptotic cell death using a live/dead assay. The CC531 cell line is of interest for evaluating potential new agents for improved CRLM treatment because it has been established as a rodent CRLM model for sub-capsular injection into the livers of syngeneic WAG/Rij rats [5,19]. Mechanistically, CF10 is more effective than 5-FU at activating biomarkers of replication stress (e.g., pRPA and pChk1), as assessed by Western blots, and causing DNA double-strand breaks (DSBs), consistent with the established TS/Top1 dual-targeting mechanism. CF10 was very well tolerated in vivo and intra-venous doses that delivered equivalent fluoropyrimidine content as an established dose of 5-FU in rats (50 mg/kg [20]) did not cause weight loss or systemic toxicities. CF10 demonstrated improved antitumor activity relative to an equivalent dosing of 5-FU; moreover, when combined with a favorable toxicity profile based on a lack of weight loss, further therapeutic advantages could be achieved with more aggressive dosing. Our findings are consistent with CF10 and CF10-based combination therapies having the potential to improve outcomes for CRLM patients relative to 5-FU-based therapies through distinct mechanistic and pharmacologic properties.

## 2. Materials and Methods

### 2.1. Cell Culture and Reagents

The rat colon carcinoma cell line CC531 was derived from WAG/Rij rats exposed to 1,2-dimethyl hydrazine. CC531 cells obtained from Dr. Sarah White (University of Wisconsin—Milwaukee) were cultured in Dulbecco’s modified Eagle medium (DMEM) (Life Technologies, Carlsbad, CA, USA) and supplemented with 10% fetal bovine serum in culture flasks at 37 °C in a humidified atmosphere containing 5% CO_2_. CF10 was obtained from ST Pharma and was validated by high-resolution mass spectrometry and was dissolved in 0.9% sterile saline. Clinical-grade 5-FU (50 mg/mL) was purchased from the Baptist Hospital clinical pharmacy. 5-FU concentrations were calculated based on the dilution of the established stock concentration. CF10 concentrations were matched to deliver equivalent nucleoside content based on UV absorbance at 260 nm. A modified clonogenic assay was used to assess the potency of CF10 and 5-FU in CC531 cells. CC531 cells were plated in 24-well plates, and, after 24 h, they were treated with the indicated concentration of CF10 or 5-FU for 72 h. Following drug treatment, the media were replaced, and cells were allowed to grow for 7 days. Cell proliferation was evaluated using the Aqueous One (Promega) reagent, in line with the manufacturer’s instructions, after the cell solution was transferred to 96-well plates.

### 2.2. Western Blotting

Proteins were isolated and their differential expressions were analyzed by Western blot, as described previously [21,22]. Briefly, the cells were lysed using RIPA buffer (50 mM Tris HCl at pH 7.4, 150 mM NaCl, 1% Triton X-100 or NP-40, 0.5% sodium deoxycholate, 0.1% SDS, 1 mM EDTA, and 10 mM NaF freshly supplemented with protease and phosphatase inhibitors). Protein concentrations were quantified using a Bradford assay (Bio-Rad), and the samples were normalized for equal loading. The samples were then heated to 100 °C for 10 min in the presence of 6× Laemmli buffer (Boston BioProducts; Milford, MA, USA). The proteins were resolved by SDS-PAGE and their expression was analyzed following immunoblotting using specific antibodies. The following antibodies were used in this study: FANCD2 (R&D MAB93691), pCHK2-T68 (Invitrogen PA5-104715), pRPA 32-S33 (Cell Signaling 10148s), pCHK1-S296 (Cell Signaling 90178s), pH2AX-S139 (Millipore 05-636), GAPDH (Santa Cruz 32233), and Vinculin (Cell Signaling 13901S).

### 2.3. Apoptosis Analysis with Flow Cytometry

CC531 cells underwent 48 h and 72 h treatments with a dose of CF10 that was 1 log less than the 5FU dose. After the treatment, the samples were analyzed for apoptosis using the Invitrogen™ eBioscience™ Annexin V-FITC Apoptosis Detection Kit (catalog number 509299), as described previously [23]. Following drug treatment, the cells were trypsinized and then stained with 5 μL of Annexin-V for 10 min at room temperature. After this, the samples were washed with binding buffer (following the manufacturer’s instructions) and incubated with 10 μL of propidium iodide (PI, 20 µg/mL) for 30 min at 37 °C. Finally, the samples were analyzed with flow cytometry for the apoptotic cells using a BD Accuri (BD Biosciences, Franklin Lakes, NJ, USA). A total of three independent experiments were performed for each time point.

### 2.4. Liver Tumor Cell Inoculation to Simulate CRLM Formation

All animal studies were undertaken in accordance with the protocols approved by the Institutional Animal Care and Use Committee at Wake Forest University School of Medicine (Winston-Salem, NC, USA). Immunocompetent WAG/Rij rats weighing 250–300 g were used because this strain is syngeneic with the CC531 colon carcinoma cell line. WAG/Rij rats are not commercially available in the U.S. and were obtained from Dr. Sarah White (UW-M) and bred at WFUSM for these studies. The rats had a controlled climate and light cycles, and all had free access to a standard laboratory diet and water. For the inoculation procedure, anesthesia was obtained using 2–3% isoflurane and then maintained at 1.5% for surgery. A 2 cm midline incision was made, and both the left and right hepatic lobes were mobilized. The CC531 cells were suspended at a density of 2 × 10^6^ cells in 200 uL and 25 uL of cell suspension, mixed 1:1 with Matrigel, and injected via a 29-gauge needle into the subcapsular portion of one of the previously specified hepatic lobes. When the needle was withdrawn, a cotton swab was used to press on the puncture site to achieve hemostasis. Once hemostasis was achieved, the wound was washed with saline, the muscle layer was closed using a 6-0 Vicryl suture, and the skin was closed using staples. An abdominal bandage was applied and the rats were allowed to recover from anesthesia before returning to the animal facility. The tumors were allowed to grow for 7 days and fluorescence imaging was performed using an RGD peptide Cy5.5 conjugate to establish tumor formation prior to initiating treatments. 

### 2.5. Treatment and Fluorescence Imaging of Tumor Progression

Tumor formation in the liver was established using fluorescence imaging following the tail vein injection of a Cy5.5-labeled cyclic “RGD” peptide (c[fK(Cy5.5)RGD]; UNC peptide synthesis facility, Chapel Hill, NC, USA) targeting integrin a_v_b_3_ (Figure S1) 10 nmol of peptidein 100 uL of 0.9% sterile saline were injected 4–24 h prior to imaging [24]. Attempts to label CC531 cells with luciferase using viral transduction were successful (Figure S2); however, we were not able to follow tumor progression in vivo afterwards by conducting bioluminescence imaging because imaging was negative for luciferase signal, even though the autopsy of rats revealed tumor formation and progression in the liver. It is likely that the luciferase-labeled CC531 cells were selected against during tumor progression in the immunocompetent host. We experienced similar issues detecting luciferase-transduced MC38 mouse CRC cells in a C57BL6/MC38 syngeneic orthotopic mouse model, and, in that model, the immunohistochemistry of liver tumor correlated with RGD peptide fluorescent signals. The fluorescence imaging of tumors was undertaken using an IVIS Lumina LT Series III system and the rats were imaged under isoflurane anesthesia. All images were analyzed using Living Image software (v4.7.4.) with an identical size region of interest for each rat. To assess the therapeutic efficacy of CF10 and 5-FU, groups of *n* = 5 rats with similar initial tumor levels based on fluorescence imaging (Figure S3) were treated with either 5-FU (50 mg/kg) or CF10 doses to deliver equivalent nucleoside content based on UV absorbance at 260 nm. The 5-FU dose is based on similar studies in the related Sprague–Dawley rat strain [20]. Rats in each group were treated 1×/week for 4 weeks. One week after the fourth treatment, all rats underwent a final imaging procedure and were then euthanized, and their livers were extracted and imaged ex vivo.

### 2.6. Statistical Analysis

All statistical analyses were conducted using GraphPad Prism 10.2.0. Experiments were conducted in triplicate ± SEM. For all in vitro experiments, five biological replicates were used for analysis, and appropriate one-way ANOVA tests with recommended Tukey corrections were conducted, with *p* < 0.05 indicating significance.

## 3. Results

**Figure 2:**
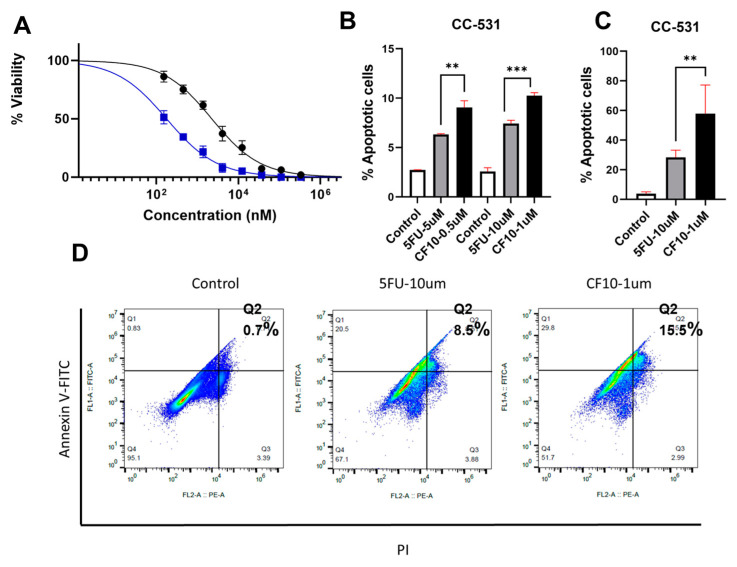
CF10 is potent in CC531 cells and induces apoptosis more efficiently than 5-FU. (**A**) Dose–response graphs for 5-FU (black) and CF10 (blue) in CC531 cells determined using a modified clonogenic assay with 48 h treatment (*N* = 4). (**B**–**D**) Graphs of % apoptotic CC531 cells based on Annexin V staining and flow cytometry analysis following the indicated treatment for (**B**) 48 h and (**C**) 72 h cells. (**D**) Representative images from flow cytometry analysis (72 h treatment) with quantification showing increased late apoptotic (Q2; AnnexinV^+^/PI^+^) cells for CF10 with a 10-fold lower concentration than 5-FU. ** *p* < 0.01, *** *p* < 0.0001.

CF10 inhibits CC531 clonogenic survival and induces apoptosis. We previously demonstrated improved potency for CF10 relative to 5-FU in the NCI60 cell line screen [18,25] and a more potent decrease in clonogenic survival for human CRC [18] and pancreatic ductal adenocarcinoma (PDA) cell lines [26] relative to 5-FU, which correlated with improved antitumor activity in xenograft models. We performed a modified clonogenic survival assay to test if the CC531 rat CRC cell line was responsive to CF10 and observed improved potency relative to 5-FU (Figure 2A). We then investigated if CF10 efficiently induced apoptotic cell death based on flow cytometry analysis for Annexin V cell surface expression and permeability to propidium iodide (PI). The percentage of early apoptotic (Annexin V^+^/PI^−^) and late apoptotic (Annexin V^+^/PI^+^) cells were increased upon CF10 treatment relative to a 10-fold higher concentration of 5-FU at 48 h (Figure 2B) and 72 h (Figure 2C). Thus, we found that CC531 cells are relatively more responsive to CF10 than 5-FU and that CF10 is a potent inducer of apoptotic cell death in this cell line.

### CF10 Causes Replication Stress and DNA DSBs in CC531 Cells

**Figure 3:**
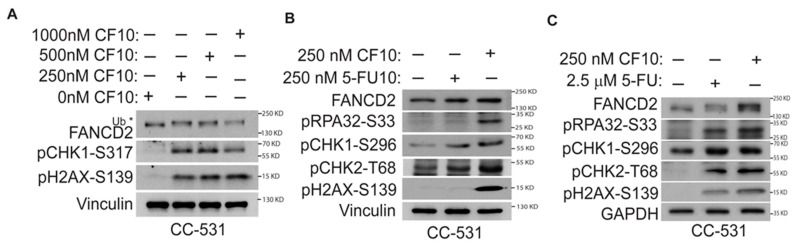
CF10 activates the ATR/Chk1 pathway, consistent with replication stress, and causes DNA DSBs in CC531 cells. Western blots from CC531 cell lysates: (**A**) CF10 dose–response graphs demonstrate that a 250 nM dose is sufficient for ATR/Chk1 pathway activation and DNA DSBs; (**B**) equimolar 5-FU is not effective in ATR/Chk1 activation and DNA DSBs; and (**C**) a 10-fold greater 5-FU dose is relatively less effective than 250 nM CF10. * indicates mono-ubiquitnated FANCD2.

We previously established that the improved potency of CF10 relative to 5-FU in human CRC cells resulted from more efficient TS inhibition and the poisoning of DNA topoisomerase 1 (Top1) [16,17], resulting in stalled replication forks and increased replication stress [18]. Replication fork collapse may result in DNA double-strand breaks (DSBs), genomic instability, and cell death [27], which is consistent with the observed increased potency of CF10 relative to 5-FU in CC531 cells (Figure 2). The adverse consequences of replication fork collapse may be countered by ATR/Chk1 pathway activation which decreases the initiation of new replication forks and may stimulate the activation of the homologous recombination (HR)-mediated repair of DNA DSBs generated at collapsed forks [28]. CF10 significantly stimulated increased pChk1 Serine-317 (S317) levels, consistent with ATR/Chk1 pathway activation (Figure 3A). FANCD2 mono-ubiquitination and pRPA32 Serine-S33 were also increased in CF10-treated CC531 cells to a greater extent than in CC531 cells treated with 5-FU at equivalent fluoropyrimidine concentrations (Figure 3B,C), consistent with the stabilization of stalled forks and the attempted repair of collapsed forks [29,30]. Further, pH2AX-S139 and pChk2 Threonine-T68 (T68) increased with CF10 treatment, consistent with the generation of DNA DSBs during the attempted repair of collapsed forks resulting in ATM/Chk2 pathway activation (Figure 3B,C). Our results are consistent with the increased potency of CF10 relative to 5-FU in CC531 cells resulting from DNA damage associated with replication fork collapse, with the extent of damage mitigated through the activation of the ATR/Chk1 and ATM/Chk2 pathways.

*CF10 is well tolerated and efficacious in the WAG/Rij Rat CRLM model*.

**Figure 4:**
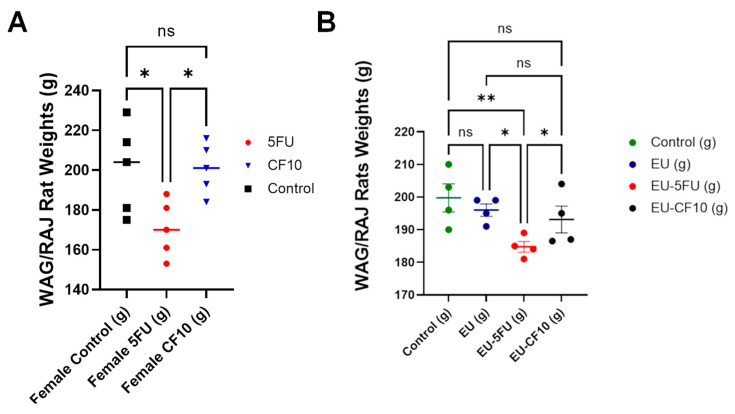
5-FU (but not CF10) induced significant weight loss in WAG/Rij rats. (**A**) The final weights of WAG/Rij rats following treatment with 5-FU (50 mg/kg), CF10 (141 mg/kg), or vehicle (0.9% sterile saline). The rats were dosed 1×/week for 4 weeks via tail vein injection. (**B**) The rats were administered a single dose of ethynyl uracil (EU; 2 mg/kg), alone or in combination with a single dose of 5-FU or CF10, as shown in (**A**). 5-FU+EU (but not CF10+EU) caused significant weight loss (* *p* < 0.05, ** *p* < 0.041, ns - not significant), Initial average weights for all groups were not significantly different in both (**A**,**B**) (Figure S4).

CRC mortality is almost exclusively from metastatic progression, with the liver being the most frequent metastatic site involved in ~50% of all CRC cases [2]. Chemotherapy with 5-FU-based combination therapy regimens is central to CRLM treatment with established survival advantages for both FOLFOX and FOLFIRI regimens [31]. However, these regimens are rarely curative and are associated with serious toxicities in many cancer patients [9] and the development of drug resistance [11], necessitating new and more effective therapies. To test if the improved potency of CF10 relative to 5-FU in CC531 rat CRC cells could translate into improved activity in a rat model of CRC liver metastasis, we evaluated both FPs using the CC531/WAGRij rat syngeneic orthotopic model. In this model, the sub-capsular injection of CC531 cells into one lobe of WAG/Rij rat liver resulted in a liver tumor mass that demonstrated the pharmacological challenges associated with chemotherapy administration for CRLM treatment [5]. 5-FU was dosed at 50 mg/kg, a dose previously used in rats [20], and i.v. tail vein injection 1×/week for 4 weeks resulted in significant weight loss (Figure 4A). However, the identical dosing of CF10 to deliver equivalent fluoropyrimidine based on UV A260 absorbance was not associated with weight loss, while initial weights were similar for all groups (Figure S4). To further investigate potential toxicity differences between CF10 and 5-FU related to altered metabolism, we tested the effects of the co-administration of ethynyl uracil (EU), an agent that inhibits the hepatic degradation of 5-FU with the dihydropyrimidine dehydrogenase (DPD) enzyme [32]. A single dose of 5-FU at 50 mg/kg resulted in significant weight loss upon co-administration with EU (2 mg/kg); however, the co-administration of CF10 dosed to deliver equivalent fluoropyrimidine content did not cause significant weight loss upon co-administration with EU. The results demonstrate that CF10 is relatively well tolerated in WAG/Rij rats and, consistent with this, is not undergoing hepatic degradation to the same extent as 5-FU.

**Figure 5:**
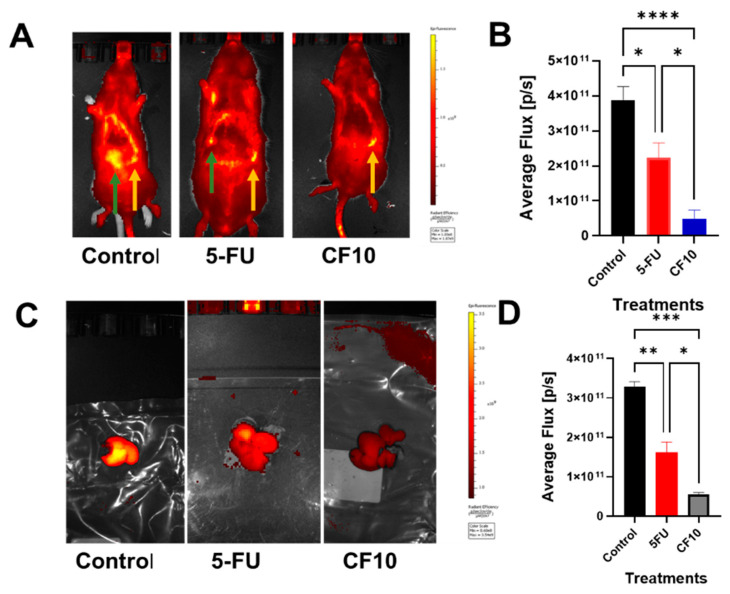
Imaging CRLM progression in WAG/Rij rats after 4 weeks of the indicated treatment, showing that CF10 was found to be effective in a rat CRLM model. Liver metastases were formed by injecting CC531 rat CRLM cells into the left hepatic lobe. Liver tumor masses were detected using a fluorescent (Cy5.5-labeled) RGD peptide and were similar in all rats at baseline (2 weeks after receiving an injection). Rats (*n* = 5 per group) were then treated for 4 weeks (1×/week via i.v. dose) with vehicle, 5-FU (50 mg/kg), or CF10 (identical to 50 mg/kg 5-FU dose based on UV A_260_). (**A**) The fluorescence imaging of rats 4 weeks after receiving treatment. Green arrows point to liver tumor signal, which is quantified in (**B**). Yellow arrows point to spleen autofluorescence. (**C**) Rats were then euthanized, and their livers were extracted and imaged ex vivo with Cy5.5 signal quantified in (**D**). Rats treated with CF10 displayed significantly reduced signals from CRLMs relative to both 5-FU and vehicle. * *p* < 0.01; ** *p* < 0.011; *** *p* < 0.015; **** *p* < 0.002.

We then evaluated the potential of CF10 to reduce the progression of liver tumor mass in the CC531 WAG/Rij rat syngeneic orthotopic CRLM model (Figure 5). As described previously [5,19], the subcapsular injection of CC531 rat CRC tumor cells into a hepatic lobe results in a liver-specific tumor mass that models CRLM for evaluating novel therapies. Tumor masses were identified using a fluorescent RGD peptide that binds to integrin α_v_β_3_ [24], as luciferase expression (Figure S2) was not maintained in CC531 cells in vivo following tumor formation. The establishment of a tumor was validated in all rats based on the detection of fluorescent signals in the liver (Figure S3). Parallel studies in a mouse CRLM model demonstrated that fluorescent imaging with the RGD peptide accurately reflected the extent of tumor progression, as assessed by histopathology. The rats were treated with either vehicle (0.9% sterile saline), 5-FU (50 mg/kg), or CF10. The CF10 dose was matched to deliver fluoropyrimidine content equivalent to the 5-FU dose based on UV A_260_ absorbance. The rats were dosed 1× per week for 4 weeks via tail vein injection. One week following the final treatment, all rats underwent a final in vivo imaging procedure (Figure 4A,B), after which the rats were humanely euthanized and their livers were extracted and imaged ex vivo (Figure 5C,D). Ex vivo liver imaging confirmed strong fluorescent signals throughout the liver for all rats in the control group, while for rats treated with 5-FU, fluorescent signals were primarily associated with a single lobe and were of significantly lower intensity relative to vehicle, consistent with the idea that 5-FU activity is used for inhibiting the progression of liver metastatic disease. Rats treated with CF10 did not have a detectable fluorescent signal in the liver in the final in vivo imaging and ex vivo imaging procedures, which did not indicate that any lobe displayed increased fluorescence intensity relative to background, a result consistent with the regression of liver tumor mass with CF10 treatment. The data are consistent with the notion that CF10 is more effective than 5-FU for the treatment of liver metastatic CRC in a rat model that replicates pharmacological challenges associated with CRLM treatment.

## 4. Discussion

The principal cause of CRC mortality is the progression of metastatic disease, with the liver being the most frequent site for CRC metastasis [2,3]. Chemotherapy is central to the management of CRC liver metastatic disease (CRLM) and 5-FU-based combination chemotherapy regimens (including FOLFOX and FOLFIRI) have contributed to improved overall survival rates for mCRC patients in the last decade. While an increasing number of CRLM patients are surviving to receive second- and even third-line treatments with regimens that include FP drugs [31], the 5-year survival rate for mCRC remains dismal at <14% [33]. Further, FP-based chemotherapy is associated with serious systemic toxicities that limit patients’ quality of life. Thus, developing the next generation of FP therapies that preserve and enhance the strong anti-tumor activity of this class of drugs, particularly in gastrointestinal malignancies, even while reducing systemic toxicities, is an important research objective. In this study, we highlighted the improved activity of CF10 (Figure 1), a second-generation fluoropyrimidine polymer that is advancing towards clinical development based on the promising anti-cancer activity of FP polymers in multiple preclinical models [14,15,26], including in an orthotopic mouse model of primary colon cancer [18]. Importantly for CF10 clinical translation, we demonstrated significantly improved antitumor activity relative to equivalent 5-FU dosing in a syngeneic orthotopic rat model that replicates pharmacological challenges associated with the treatment of liver metastatic disease. Our results demonstrate that CF10 displays pharmacological properties suited for the effective treatment of liver metastatic disease. Further, strong activity was achieved without significant weight loss or other indications of systemic toxicities, while 5-FU administration was associated with significant weight loss in this rat model. 

Mechanistically, CF10’s substantial potency advantage relative to 5-FU in CRC cells [16,25] is associated with increased replication stress and the activation of apoptosis. We demonstrated that CF10 was more potent than 5-FU to the rat CRC cell line CC531 in a clonogenic assay (Figure 2A), and a 10-fold lower concentration of CF10 resulted in a significantly greater percentage of late apoptotic cells at a 72 h timepoint (Figure 2B–D). In previous studies, we demonstrated that CF10 inhibited thymidylate synthase (TS) more effectively than equivalent 5-FU concentrations, consistent with the direct release of fluorodeoxyuridylate (FdUMP), a TS-inhibiting metabolite. TS inhibition depletes CRC cells of thymidylate and consequently thymidine triphosphate, causing the slowing and stalling of replication forks. Consistent with increased replication stress, we demonstrated that CF10 stimulated the mono-ubiquitination of FANCD2, a marker of stalled replication forks (Figure 3). CF10 treatment also more effectively activated pChk1 (S296) and pRPA (S33), consistent with ATR/Chk1 pathway activation following replication fork collapse. In previous studies, we demonstrated that ATR/Chk1 inhibitors selectively enhanced F10 but not 5-FU cytotoxicity in human CRC cell lines [34], while inhibitors of the ATR/Chk1/Wee1 pathway enhanced CF10 but not 5-FU cytotoxicity in pancreatic ductal adenocarcinoma [35]. The repair of stalled replication forks may proceed via the formation of Mus81-induced DNA DSBs [36] and our studies demonstrated that CF10 more efficiently induced pH2AX-S139 and activated the ATM/Chk2 pathway relative to 10-fold greater 5-FU concentrations (Figure 3). Our results are consistent with increased potency for CF10 relative to 5-FU through increased replication stress.

The efficacy of FP-based chemotherapy for the treatment of mCRC and CRLM is countered by the serious systemic toxicities that occur in many patients treated with these drugs [9]. In this study, we dosed rats with 5-FU at 50 mg/kg 1×/week for 4 weeks and, consistent with previous studies, observed significant weight loss with this dose [20]. In contrast, equivalent dosing with CF10 did not cause any weight loss (Figure 4A). The causes of 5-FU-induced GI-toxicity are not definitively known; however, previous studies demonstrated that 5-FU-induced intestinal damage and cell death in mice were reversed by uridine co-treatment, consistent with GI toxicity originating (at least in part) from RNA-mediated effects [37]. Thus, the improved toxicity profile for CF10 is consistent with its more direct conversion to fluorodeoxyuridylate and consequently reduced levels of RNA-directed metabolites that are primarily responsible for GI toxicity. The systemic toxicities of 5-FU are particularly severe in individuals with deficiencies in DPD activity that are associated with SNPs that occur in up to 5% of humans [9]. In these individuals, 5-FU is not degraded efficiently in the liver, resulting in relatively higher levels in tissues, including in the GI tract. To model these effects, we performed studies using the DPD inhibitor 5-ethynyluracil (EU) [32]. A single dose of 5-FU (50 mg/kg) caused significant weight loss in WAG/Rij rats when combined with EU (2 mg/kg). In contrast, the combination of CF10+EU delivering equivalent FP and equivalent EU did not cause significant weight loss in rats (Figure 4B). Our results highlight the potential for CF10 to be administered at relatively higher doses than is possible with 5-FU, potentially further increasing its anticancer activity and its therapeutic advantages relative to 5-FU. Further, patients with DPD deficiency may not be as high-risk for developing serious toxicities with CF10 treatment as for 5-FU, reducing the expense associated with the increased length of hospitalization for many DPD-deficient patients.

## 5. Conclusions

In this study, we demonstrated improved anticancer activity for CF10 relative to equivalent 5-FU concentrations in a rat-based syngeneic orthotopic model of colorectal cancer liver metastatic disease (Figure 5). CRC is a leading cause of cancer-related mortality and liver metastasis is a principal cause of CRC mortality. Our results are consistent with the finding that there is strong potential for CF10 to treat patients with CRLMs more effectively than current 5-FU-based therapy. Our results support the further development of CF10 for improved CRLM treatment that may contribute to improved outcomes for this highly lethal malignancy.

## Data Availability

Data will be made available on request.

## Supplementary Materials

The following supporting information can be downloaded at: https://www.mdpi.com/article/10.3390/cancers16071360/s1. Figure S1: Structure and analytical data for cyclic RGD peptides; Figure S2: Validation of luciferase expression in CC531-luc cells; Figure S3: Fluorescent imaging of rats prior to treatment; Figure S4: Initial weights of rats prior to treatment.
