# Supplementary material for: Enhanced Therapeutic Efficacy of the Nanoscale Fluoropyrimidine Polymer CF10 in a Rat Colorectal Cancer Liver Metastasis Model
Source: Cancers (Basel). 2024 Mar 30;16(7):1360. doi: 10.3390/cancers16071360 (PMC11011147; doi:10.3390/cancers16071360)
Supplement: Supplementary file 1 [file cancers-16-01360-s001.zip › cancers-2932584-supplementary.pdf]

Supplementary Material:

P4409v2 I, **c[fK(Cy5.5)**RGD**]**, 1169.46, HPLC purity 99%, 4.07 mg
MS ok (585.0 Da [M+H]++)

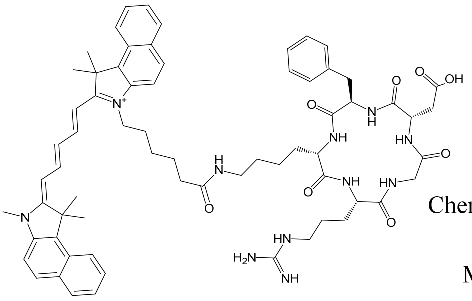

Chemical Formula: $C_{67}H_{82}N_{11}O_8^{+}$
Exact Mass: 1168.63
Molecular Weight: 1169.46

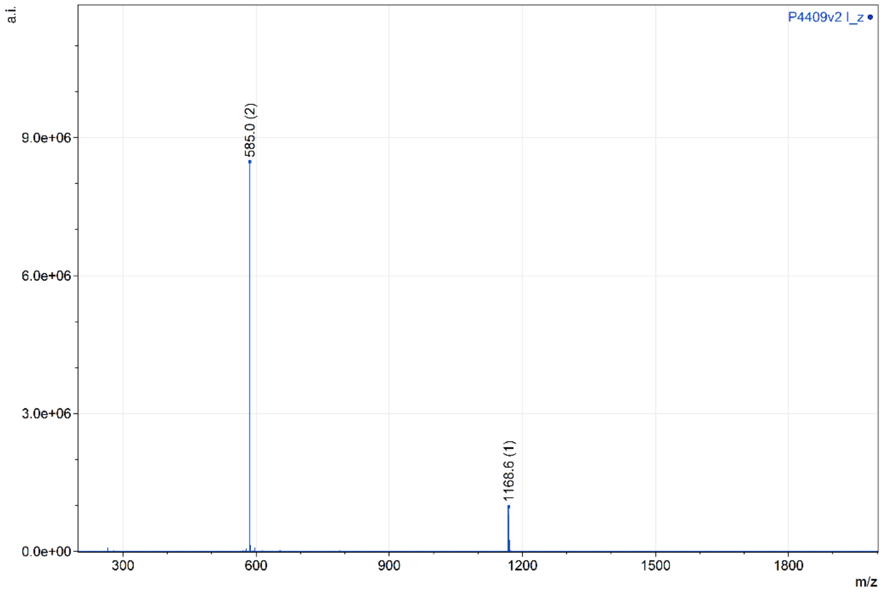

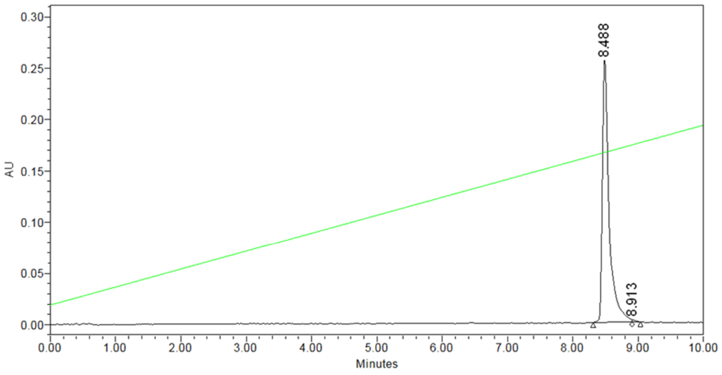

	RT	% Area	Area	Height
1	8.488	99.62	1837390	257499
2	8.913	0.38	6993	2308

Figure S1. Structure and analytical data for cyclic RGD peptide. Peptide was labeled with Cy5.5 for tumor imaging in the CC531/WAGRij rat model. Shown are mass spectrometry identification (middle) and HPLC validation of peptide purity (bottom).

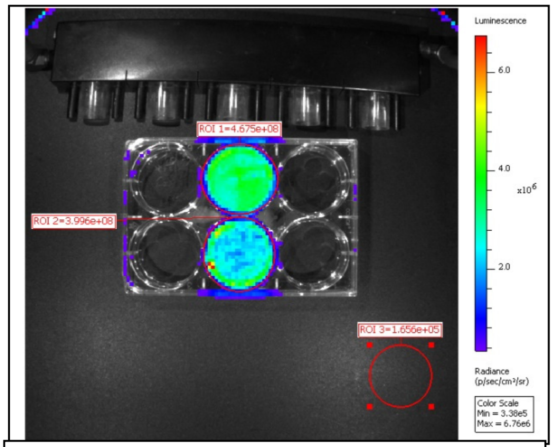

Figure S2. Validation of luciferase expression in CC531-luc cells. The cells robustly express luciferase *in vitro*.; however, expression was lost *in vivo*.

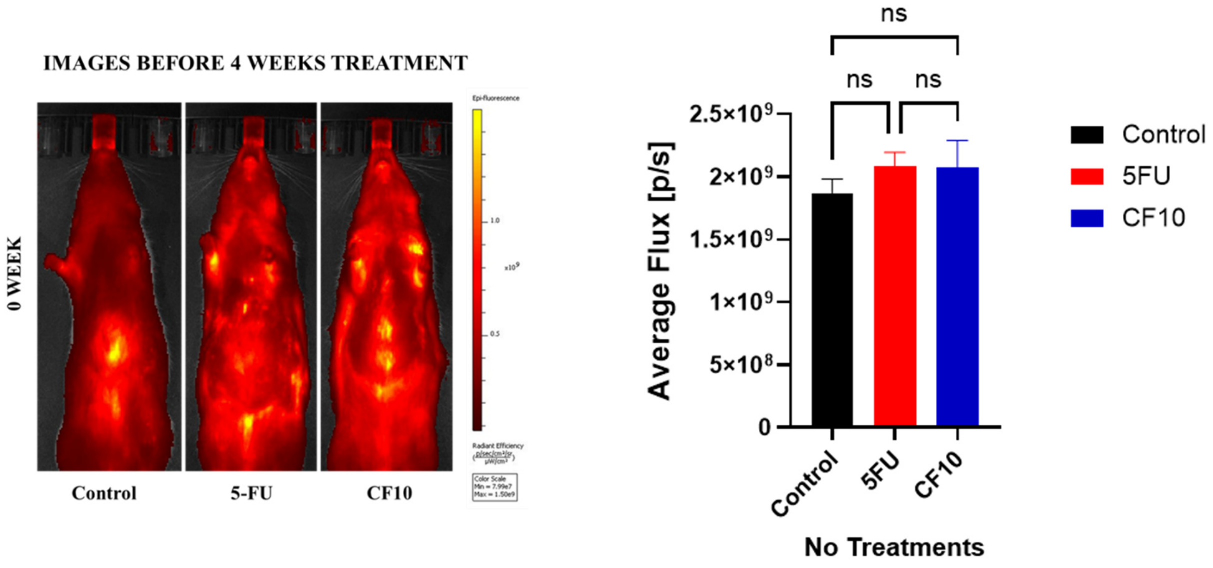

Figure S3. Fluorescent imaging of rats prior to treatment.

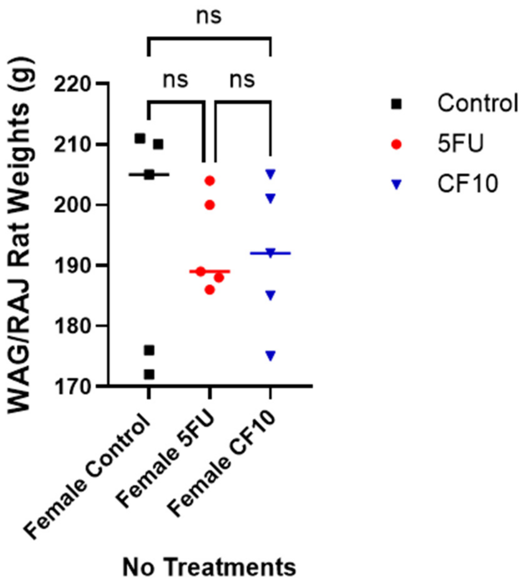

Figure S4. Initial weights for rats prior to treatment.
